# Magnetic Hyperthermia with Iron Oxide Nanoparticles: From Toxicity Challenges to Cancer Applications

**DOI:** 10.3390/nano15191519

**Published:** 2025-10-04

**Authors:** Ioana Baldea, Cristian Iacoviță, Raul Andrei Gurgu, Alin Stefan Vizitiu, Vlad Râzniceanu, Daniela Rodica Mitrea

**Affiliations:** 1 Department of Physiology, University of Medicine and Pharmacy, Clinicilor 1, 400006 Cluj-Napoca, Romania; ioana.baldea@umfcluj.ro (I.B.); gurgu.raul.andrei@elearn.umfcluj.ro (R.A.G.); vizitiu.alin.stefan@elearn.umfcluj.ro (A.S.V.); razniceanu.vlad@elearn.umfcluj.ro (V.R.); rodica.daniela.mitrea@umfcluj.ro (D.R.M.); 2 Department of Pharmaceutical Physics-Biophysics, Faculty of Pharmacy, “Iuliu Hatieganu” University of Medicine and Pharmacy, 6 Pasteur St., 400349 Cluj-Napoca, Romania

**Keywords:** magnetic hyperthermia, iron oxide nanoparticles, cancer therapy, immunotherapy, clinical translation

## Abstract

Iron oxide nanoparticles (IONPs) have emerged as key materials in magnetic hyperthermia (MH), a minimally invasive cancer therapy capable of selectively inducing apoptosis, ferroptosis, and other cell death pathways while sparing surrounding healthy tissue. This review synthesizes advances in the design, functionalization, and biomedical application of magnetic nanoparticles (MNPs) for MH, highlighting strategies to optimize heating efficiency, biocompatibility, and tumor targeting. Key developments include tailoring particle size, shape, and composition; doping with metallic ions; engineering multicore nanostructures; and employing diverse surface coatings to improve colloidal stability, immune evasion, and multifunctionality. We discuss preclinical and clinical evidence for MH, its integration with chemotherapy, radiotherapy, and immunotherapy, and emerging theranostic applications enabling simultaneous imaging and therapy. Special attention is given to the role of MNPs in immunogenic cell death induction and metastasis prevention, as well as novel concepts for circulating tumor cell capture. Despite promising results in vitro and in vivo, clinical translation remains limited by insufficient tumor accumulation after systemic delivery, safety concerns, and a lack of standardized treatment protocols. Future progress will require interdisciplinary innovations in nanomaterial engineering, active targeting technologies, and real-time treatment monitoring to fully integrate MH into multimodal cancer therapy and improve patient outcomes.

## 1. Introduction

As one of the leading global health challenges, malignant diseases remain a major cause of mortality, primarily arising from the progressive accumulation of genetic mutations in normal cells that promote unchecked cell division and tumor development. Despite decades of research and therapeutic advances, the overall mortality rates for several cancer types have only modestly declined [1]. Conventional cancer treatment strategies typically include surgical resection, radiotherapy, and systemic therapies such as chemotherapy, hormonal therapy, and targeted biological agents [2]. While these modalities have demonstrated clinical efficacy, they are frequently associated with significant adverse effects. Surgical interventions, for instance, can result in postoperative complications, tissue damage, and the potential dissemination of malignant cells, thereby increasing the risk of metastasis [3]. Chemotherapy involves the administration of cytotoxic agents that often lack tumor specificity, leading to collateral damage to healthy tissues and systemic toxicity [4]. Similarly, radiotherapy employs ionizing radiation which, when applied over extended periods, may compromise the structural and functional integrity of surrounding normal tissues [5].

Hyperthermia treatment, or thermotherapy, has emerged as a promising adjunct or alternative to conventional treatments. This approach involves elevating the temperature of body tissues to induce cancer cell apoptosis while sparing normal cells [6]. The therapeutic application of heat dates back to ancient civilizations, including those of Greece, Egypt, Rome, and India [7]. In the 19th century, spontaneous tumor regression in febrile patients was documented, prompting early investigations into hyperthermia for oncological applications, such as in the treatment of cervical cancer [7,8]. Since the 1970s, hyperthermia has garnered renewed clinical interest, with controlled trials exploring its efficacy in cancer treatment. Studies have demonstrated that cancer cells are more susceptible to temperatures between 42 and 45 °C, undergoing apoptosis, whereas normal cells exhibit greater thermal resilience [9].

Depending on the tumor’s location, depth, and stage of progression, three main hyperthermia strategies have been established for clinical application: local, regional, and whole-body hyperthermia. Whole-body hyperthermia is typically employed in cases involving deep-seated tumors or disseminated metastases, where the entire body is uniformly heated using methods such as hot water baths, thermal chambers, and infrared radiation [10,11,12,13]. For advanced-stage malignancies confined to specific areas, regional hyperthermia is applied through techniques like thermal perfusion, external applicators, and microwave antennas to deliver targeted heat [10,11,12,13]. Local hyperthermia, the least invasive approach, is primarily used for treating localized tumors situated either superficially or within accessible body cavities.

Over the past century, substantial technological progress has led to the development of magnetic nanoparticles (MNPs), which have attracted growing interest in biomedical research, particularly in oncology. These nanomaterials possess the unique ability to convert electromagnetic energy into heat when exposed to an external alternating magnetic field (AMF) [14,15]. Importantly, the penetration depth of AMF is not significantly attenuated by biological tissues, allowing effective activation of MNPs even within deep-seated tumors [15,16]. Once internalized by cancer cells, MNPs function as localized heat sources, raising the temperature of tumor tissue to levels sufficient to induce apoptosis. This approach, known as magnetic hyperthermia (MH), has emerged as a promising and innovative therapeutic strategy with the potential to enhance cancer treatment outcomes while minimizing damage to surrounding healthy tissues [16,17].

The first experimental evidence supporting the use of MH for cancer treatment was reported in 1957 by Gilchrist et al. [18], who conducted an in vitro study involving lymph nodes containing colon and rectal cancer metastases. In this pioneering work, maghemite (γ-Fe_2_O_3_) nanoparticles (NPs), ranging in size from 20 to 100 nm, were introduced into the lymph nodes and subjected to an AMF with an amplitude (H) of 16–19.2 kA/m at a frequency (f) of 1.2 MHz. The resulting temperature increases to 43–46 °C successfully eradicated carcinoma cells by destroying the metastatic tissue [18]. As a result of extensive research and due to their favorable biocompatibility and biodegradability, both -γ-Fe_2_O_3_- and its reduced form, magnetite (Fe_3_O_4_), were approved by the U.S. Food and Drug Administration (FDA) for clinical trials [19]. Consequently, iron oxide nanoparticles (IONPs) have become the most widely used agents in MH applications [20,21]. The therapeutic potential of MH reached a significant milestone with the advancement of the German company MagForce AG, which received regulatory approval from the European Union to clinically treat glioma patients [22,23,24,25].

The interaction between AMFs and biological tissues generates non-specific heating through the induction of eddy currents. This can activate the body’s thermoregulatory responses and produce complex thermal gradients throughout the patient’s body [26,27]. To ensure safety, a limit has been established for human exposure to AMFs by restricting the product of H and f to a maximum of 5 × 10^9^ A·m^−1^·s^−1^ [28]. However, the heat dissipation capabilities of commercially available MNPs, such as Nanotherm, Feridex, and Resovist, remain inadequate within physiologically safe AMF parameters. As a result, the therapeutic effect is often insufficient for complete tumor ablation, limiting the widespread adoption of MH as a standalone treatment option in clinical settings.

To advance the clinical application of MH in cancer therapy, two principal strategies have been identified in scientific literature. The first approach focuses on the design and synthesis of MNPs with enhanced intrinsic magnetic properties, such as increased saturation magnetization (M_s_) and magnetic anisotropy (K), to improve heat generation efficiency even at low concentrations. This can be achieved by manipulating parameters such as NP size, shape, chemical composition, and surface morphology. As these aspects have been extensively covered in previous reviews [29,30,31], we will therefore briefly summarize the most significant developments in this area in the first part of our review. Notably, numerous studies have reported that only a small fraction of systemically administered MNPs effectively accumulate at the tumor site [32]. Consequently, clinical efficacy often relies on direct intratumoral injections, which restrict treatment to tumors that are accessible [33]. To address this limitation, recent research has increasingly focused on combining MH with other anticancer modalities within a single, multifunctional MNP-based nanoplatform [34]. The aim of this review is to highlight the advancements and the challenges that the MH faces from the design of MNPs to ensure biocompatibility, tumor specificity, and clean biodegradation in the living organisms, while still preserving the hyperthermic and drug delivery properties, towards the biological testing on different in vitro and in vivo models and eventually clinical patients, respectively. This integrative strategy represents the second major approach in the field and will be explored in detail in the second part of this review. Clinical advancements in the field, based on clinical studies of MH efficacy in oncologic patients, will be further discussed in the third part of this review. Finally, the challenges, outlook and conclusions regarding the advances of MH in oncological research and the prospects of MH to become an important adjuvant therapy in the oncologic patients are described.

## 2. Strategies to Enhance Magnetic Heating Efficiency of Magnetic Nanoparticles

The thermal effect generated by MNPs under AMF stimulation is quantified by a physical parameter known as the specific absorption rate (SAR), also referred to as specific loss power (SLP). SAR represents the amount of heat released per unit time per unit mass of MNPs and is typically expressed in watts per gram (W/g) [35]. The SAR value is influenced by both the intrinsic properties of the MNPs—such as NPs volume and M_s_—and the extrinsic parameters of the applied AMF. To enhance the induction heating performance, efforts have been directed toward optimizing the intrinsic magnetic properties of MNPs. Simultaneously, thermal efficiency has been externally improved by increasing the frequency and amplitude of the AMF in different MH setups.

### 2.1. Formulation

For biomedical applications requiring injectable nanoprobes, superparamagnetic (SP) NPs—commonly referred to as SPIONs (superparamagnetic iron oxide nanoparticles)—are generally preferred. Their lack of remanent magnetization (M_r_) in the absence of an external magnetic field facilitates colloidal stability, enhances dispersion in biological fluids, and minimizes the risk of particle aggregation. Among these, ultra-small SPIONs (typically < 5 nm in core diameter) have emerged as promising candidates for magnetic resonance imaging (MRI) contrast enhancement due to their excellent magnetic relaxation properties [36,37]. However, in the context of MH, such ultra-small SPIONs often exhibit low SAR values, limiting their heating efficiency [38]. Furthermore, a significant reduction in heating performance is commonly observed when SPIONs are internalized into cells or embedded in tissues, likely due to restrictions in Brownian motion and changes in local viscosity [39]. As a result, considerable research efforts have been directed toward optimizing NPs properties within the SP regime to enhance the efficacy of MH under physiological conditions.

The magnetic properties of MNPs are strongly influenced by their size. An increase in the size or volume of MNPs typically results in a higher M_s_, which reflects the net alignment of all magnetic spins within the NPs. This increase continues up to a critical threshold, beyond which M_s_ stabilizes and approaches the bulk material value. Numerous studies have reported a significant increase in the SAR with the growth of spherical SPIONs, ranging from several tens to several hundreds of W/g [40,41,42,43,44,45]. As the diameter of MNPs increases, their magnetic anisotropy energy—the energy responsible for maintaining the magnetic moment in a preferred orientation—also increases. For each MNP composition, there exists a characteristic size at which the anisotropy energy surpasses the thermal energy, stabilizing the magnetic moment along a preferred axis known as the easy axis of magnetization. This transition drives MNPs from a SP to a ferromagnetic regime, characterized by the appearance of hysteresis loops. These loops exhibit M_r_, representing the residual magnetization at zero external field, and coercivity (H_c_), the magnetic field required to bring the magnetization to zero. The magnetic MH efficiency of MNPs is governed by their dynamic hysteresis behavior [46], which is influenced not only by Neel and Brownian relaxation mechanisms but also by DC magnetic hysteresis. As a result, SAR values in ferromagnetic NPs can be nearly an order of magnitude higher than in their SP counterparts [47,48,49,50]. However, despite their high heating efficiency, ferromagnetic NPs are generally less suitable for biomedical applications due to their colloidal instability and finite coercive field, which promote aggregation and reduce biocompatibility [51,52,53].

Individual SPIONs often exhibit limited magnetic moments, which restrict their efficiency in MH. However, when these SPIONs are organized into clusters through self-assembly or aggregation, magnetic interactions between the closely packed cores can induce collective magnetic behaviors, resulting in enhanced net magnetic moments [54]. This clustering significantly improves key magnetic properties, such as M_s_ and magnetic susceptibility, thereby increasing their responsiveness to external magnetic fields. Moreover, the clustered architecture provides improved colloidal stability and resistance to uncontrolled aggregation, preserving SP behavior while ensuring long-term performance under physiological conditions [55,56]. Cluster formation can occur via two primary strategies. In a two-step process, SPIONs are first synthesized as discrete particles, followed by their assembly into clusters mediated by ligand-induced colloidal interactions, such as hydrophobic or electrostatic forces [57]. Alternatively, clustering can occur in a single-step synthetic route, wherein the NPs aggregate during formation [58,59]. The polyol method has been widely employed for this purpose in the past decade due to its adaptability, scalability, and ability to control NP morphology. This method enables the formation of various hierarchical structures, including nanoclusters, nanoflowers, and hollow spheres, by tuning reaction parameters such as temperature, solvent polarity, and precursor concentration [60,61,62,63,64,65]. Flower-like MNPs (nanoflowers) with coherent crystallographic orientation between cores have garnered particular attention for their superior magnetic heating performance [66,67,68]. The improved SAR values are thought to arise from collective spin dynamics and magnetic coupling effects within the multicore structure, which favor more efficient energy dissipation under AMF [69,70].

### 2.2. Shape

**Figure 1:**
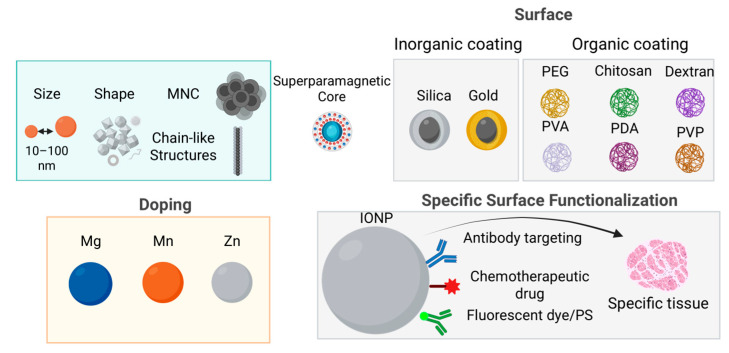
Various strategies have been developed to design efficient nanoplatforms with enhanced MH capabilities, such as increasing size and tuning the shape. Metallic doping of manganese and zinc with magnesium improves magnetic properties as well as heating capabilities. Additionally, assembling nanoparticles into magnetic nanoclusters or chain-like structures can further amplify their hyperthermic effects. Another approach involves either inorganic or organic surface coatings, also applied to improve biocompatibility, facilitate cellular uptake, and provide functional binding sites, using silica, gold, organics such as synthetic polymers (such as polyethylene glycol (PEG), polyvinyl alcohol (PVA), polydopamine (PDA), or polyvinylpyrrolidone (PVP)) or natural polymers (such as chitosan and dextran). These nanoplatforms can also be functionalized to achieve theranostic capabilities by modifying the shell or polymer coating. Ligands (e.g., folic acid or antibody fragments) can be added for specific targeting of tumor cells, while therapeutic agents (such as chemotherapeutic drugs) enable combined MH and chemotherapy. For imaging purposes, fluorescent dyes may be incorporated to enhance tumor visualization, together with MRI. Moreover, photosensitizers can be included to facilitate combined photodynamic or photothermal therapy alongside MH.

In the case of MNPs, surface atoms represent a significant proportion of the total atomic content, and their magnetic and chemical behavior often diverges from that of the bulk material. This is primarily due to the intrinsically high surface-to-volume ratio of MNPs, which causes surface effects to dominate their overall magnetic properties. Notably, the asymmetric coordination of surface atoms gives rise to spin disorder or spin canting, ultimately reducing the M_s_ of the NPs [71]. This phenomenon is especially pronounced in spherical SPIONs, which expose multiple crystallographic facets with numerous edges and corners [72]. These structural features enhance surface anisotropy and require greater energy to reorient surface magnetic moments, negatively impacting their heat dissipation efficiency under AMF. Consequently, the synthesis of anisotropic SPIONs has gained considerable interest as a strategy to enhance MH performance [73]. Various non-spherical morphologies, including nanocubes [74,75,76,77,78], octopods [79], octahedrons [80,81], nanorods [82,83,84], nanodiscs [85], nanorings [86], and polyhedral structures [87], have demonstrated improved heating efficiency compared to their spherical counterparts (Figure 1).

### 2.3. Doping with Metallic Ions

Fe_3_O_4_ exhibits an inverse cubic spinel structure, in which O^2−^ anions form a face-centered cubic (FCC) lattice that accommodates two distinct cationic sublattices: tetrahedral (A) sites, exclusively occupied by trivalent iron ions (Fe^3+^), and octahedral (B) sites, shared by both divalent (Fe^2+^) and trivalent (Fe^3+^) cations. Superexchange interactions mediated by O^2−^ anions govern the magnetic coupling between these cations, resulting in three main interaction types: A–O–A, B–O–B (intra-sublattice), and A–O–B (inter-sublattice). While intra-sublattice interactions tend to be ferromagnetic, the inter-sublattice (A–O–B) interactions are antiferromagnetic in nature, giving rise to the characteristic ferrimagnetism of Fe_3_O_4_. The net magnetic moment per formula unit is determined by the difference between the magnetic moments at B and A sites (μ_oct_ − μ_tet_), primarily attributed to the presence of Fe^2+^ ions on B sites, resulting in a net moment of approximately 4 μ_B_ (Bohr magnetons) per formula unit.

Tailoring the magnetic properties of Fe_3_O_4_ NPs, can be effectively achieved through substitutional doping of Fe^2+^ ions with other divalent transition metal cations such as Mn^2+^, Co^2+^, Ni^2+^, Cu^2+^, and Zn^2+^ (Figure 1). For instance, replacing Fe^2+^ (3d^6^) with Mn^2+^ (3d^5^), which possesses a higher magnetic moment, enhances the overall magnetic moment to 5 μ_B_ per formula unit. This substitution has been shown to significantly increase the M_s_, reaching values up to 110 emu/g_metal_, and consequently improve both heating efficiency in MH [88,89,90,91,92,93]. Interestingly, zinc doping—despite Zn^2+^ (3d^10^) having zero magnetic moment—also leads to modulation of M_s_ due to site-specific cation rearrangement within the spinel lattice. At low doping concentrations (x < 0.5 in Zn_x_Fe_3_–_X_O_4_), Zn^2+^ preferentially occupies A sites, displacing Fe^3+^ cations to B sites. This redistribution increases the magnetic moment at B sites (μ_oct_) and reduces that at A sites (μ_tet_), resulting in a substantial increase in M_s_ (161 emu/g_metal_), displaying approximately four times greater heating efficiency compared to conventional SPIONs [72,94,95,96]. Another example is the use of magnesium as a dopant. Mg_x_Fe_2_O_3_ nanoparticles (x = 0–0.15) with an average size of 7 nm demonstrated exceptional heating power—approximately 100 times higher than commercial Fe_3_O_4_ (Feridex)—attributed to enhanced magnetic susceptibility and ~50% octahedral Fe^3+^ vacancy occupation by Mg^2+^ ions, as supported by atomic structural modeling [97]. Although IONPs are frequently doped with the above-mentioned cations in efforts to enhance M_s_ and SAR, the resulting properties vary considerably depending on the synthesis parameters and distribution of dopants between T_d_ and O_h_ sites. Thus, precise control of dopant incorporation within the Fe_3_O_4_ lattice is essential to optimize magnetic and thermal performance.

### 2.4. Controlled Nanoscale Assembly of MNPs

It has been demonstrated that during MH experiments, the application of an AMF promotes the organization of MNPs into elongated assemblies or chains. This field-induced structuring significantly influences the SAR and overall heat generation capabilities of the MNPs [98,99,100]. Chain formation behavior has also been observed intracellularly, where MNPs internalized by cells tend to align in response to the AMF [101]. Further studies have revealed that such alignment occurs within intracellular vesicles and does not compromise cellular morphology or nuclear integrity [102]. In a related context, magnetosomes—MNPs biosynthesized by magnetotactic bacteria—exhibit superior heating efficiency compared to their synthetic analogs, primarily due to their intrinsic chain-like organization [103,104,105]. Consequently, the controlled nanoscale assembly of MNPs to enhance SAR represents an important topic in MH research.

Several groups have investigated the effect of pre-aligning MNPs in a static magnetic field (H_DC_) on their heating performance. They have demonstrated that MNPs pre-aligned under a H_DC_ before immobilization (e.g., via gelation in a solid matrix) produce significantly higher SAR values when aligned parallel to the AMF, compared to randomly oriented NPs [106,107,108,109,110]. Furthermore, two in vitro studies have shown that either culturing cancer cells with MNPs under a H_DC_ or pre-aligning incubated MNPs significantly improves MH efficiency and enhances cancer cell destruction, compared to the non-aligned scenario [111,112].

Another promising strategy for boosting MNP heating efficacy involves the superposition of a s H_DC_ on the AMF during MH treatment. Experimental evidence indicates that for SPIONs, this approach can lead to SAR enhancements of up to 40% relative to AMF-only conditions [16]. Chain formation in this system was confirmed by atomic force microscopy [113]. In contrast, ferromagnetic MNPs, when aligned under an H_DC_ in low-viscosity agar matrices (0.10–2.00 wt%), exhibit even more pronounced SAR increases—up to threefold—especially at low agar concentrations (0.1 wt%), where NPs mobility is less restricted [10]. Similarly, the application of static fields (H_DC_ = 10–20 kA/m) parallel to the AMF during MH measurements has been shown to significantly increase the SAR of zinc ferrite NPs in a concentration-dependent manner, with greater effects observed at lower particle concentrations [114].

### 2.5. Surface Functionalization

An effective strategy to enhance the SAR of SPIONs lies in tailoring their surface coating properties. Surface coatings influence both magnetic behavior and colloidal stability, ultimately impacting heating efficiency under an AMF [115].

Liu et al. investigated the influence of coating thickness on the SAR of Fe_3_O_4_ coated with phosphorylated methoxy polyethylene glycol 2000 (PEG2000). Their findings revealed that for smaller-sized NPs (e.g., 9 nm and 19 nm), SAR increased as coating thickness decreased, an effect attributed to enhanced Brownian relaxation losses. Notably, the PEGylated SPIONs retained high SAR values under various physiological conditions, indicating strong colloidal and functional stability [116].

In another study, Fe_3_O_4_ nanoparticles coated with PEG of different molecular weights demonstrated resistance to the formation of collective coatings. This prevented the agglomeration of NPs into large clusters and preserved their high SAR across environments with varying ionic strengths and viscosities, including distilled water, physiological saline, agar, and cell culture media [117]. Further investigations into the impact of surface functionalization demonstrated that hydrophilic SPIONs synthesized via oleate capping and subsequently modified with diverse ligands (PEG; dimercaptosuccinic acid—DMSA; cetrimonium bromide—CTAB; stearic acid—SA; and poloxamer 188—P188) exhibited different heating profiles [118]. Ligand exchange with PEG and DMSA promoted NP dispersion, whereas intercalation with CTAB and SA or encapsulation with P188 led to agglomeration into spherical clusters. MH experiments showed significantly higher SAR for the PEG- and DMSA-modified MNPs, emphasizing the detrimental effect of aggregation on heating performance [118]. Additionally, dextran-coated SPIONs with a diameter of 7 nm have also demonstrated high SAR values [119], supporting the notion that both organic and inorganic surface coatings can substantially enhance magnetic heating efficiency.

Inorganic coatings, such as gold or silica shells, have also proven effective [120]. Mohammad et al. [121] reported a 4–5-fold enhancement in SAR when SPIONs were coated with a thin gold shell (0.5 nm). Moreover, a maximum SAR value of 1300 W/g_Fe_ was achieved for dumbbell-shaped hybrid nanostructures comprising a 24 nm Fe_3_O_4_ domain attached to a 9 nm gold seed [122]. These structures benefit from synergistic effects between magnetic and plasmonic components, improving thermal response. Silica coating represents another widely used approach for surface functionalization of SPIONs [123,124,125]. Individual SPIONs coated with a silica shell were shown to maintain colloidal stability and avoid magnetic dipolar interactions, particularly under AMF exposure [126]. This led to superior heating performance compared to uncoated SPIONs or clusters encapsulated within a common silica shell [127,128,129,130]. These findings collectively emphasize the critical role of surface chemistry and MNPs architecture in optimizing the MH potential of SPIONs.

### 2.6. AFM Characteristics

In general, MH experiments demonstrate that SAR tends to increase with both the frequency (f) and amplitude (H) of the AMF. However, the heat released during MH cannot be indefinitely enhanced solely by tuning these two external AMF parameters, due to both biological safety limits and intrinsic MNPs properties.

First, to prevent overheating of healthy tissues due to eddy currents, safety guidelines such as the well-known Brezovich limit (H × f ≤ 5 × 10^9^ A·m^–1^·s^–1^) have been established [28]. Although some recent studies suggest that higher limits could be acceptable under certain conditions [131,132], García-Alonso et al. propose a more permissive threshold of H × f ≤ 9.6 × 10^9^ A·m^–1^·s^–1^ [133].

Second, while SAR is often assumed to scale linearly with f across various MNP types and sizes, its dependence on H is more complex. For SPIONs smaller than ~10 nm, SAR typically follows a quadratic relationship with H (i.e., SAR ∝ H^2^) [35,134,135]. In contrast, for larger NPs, SAR exhibits more complex field dependencies, sometimes deviating from simple power-law behavior [50]. Importantly, the quadratic dependence is generally observed only at low H; beyond this range, SAR tends to saturate [136]. This saturation effect has been correlated with the NP M_s_ and is supported by numerical simulations that incorporate the field dependence of both Néel and Brown relaxation times [136]. For ferromagnetic NPs, SAR saturation follows a sigmoidal trend as a function of H, a behavior that has been reported in several experimental and theoretical studies [61,87,109,114,137,138].

The saturation of SAR with increasing H, together with the safety constraints imposed by the H × f product limit, highlights a fundamental limitation of MH: the amount of heat that can be safely and effectively delivered to deep-seated tumors is inherently restricted. Consequently, MH alone is unlikely to achieve full tumor eradication, particularly in aggressive or resistant cancer types. For this reason, MH is more appropriately used as an adjuvant strategy, enhancing the efficacy of conventional treatments such as chemotherapy, radiotherapy, and immunotherapy. When applied in combination, MH can sensitize tumor cells to these therapies by promoting localized hyperthermia, thereby offering a synergistic anticancer approach [139].

### 2.7. Magnetic Nanoparticles for Combined Therapy and Imaging

The first clinical application of MNPs was as contrast agents in MRI. This technic relies on the nuclear magnetic resonance (NMR) of hydrogen protons, which constitute about 63% of the body mass. MNPs generate inhomogeneous magnetic field gradients in the surrounding medium, which directly affect the relaxation dynamics of nearby water protons, leading to accelerated dephasing. This results in increased spin–lattice and spin–spin relaxation rates (1/T_1_ and 1/T_2_, corresponding to the transverse and longitudinal relaxation times, respectively). In particular, MNPs with high M_s_ produce strong local field perturbations, thereby enhancing spin–spin relaxation, shortening T_2_ and causing signal loss (negative contrast) in T_2_-weighted images [140,141,142]. Therefore, MNPs with enhanced magnetic properties can act simultaneously as efficient mediators in MH and as effective contrast agents in MRI and vice versa. Among spherical IONPs, those with diameters between 25 and 30 nm (at the boundary of the ferrimagnetic/SP regime) exhibit maximum transverse relaxivity [110,143]. Compared to spherical IONPs, faceted, cubic, octopod, and nanorod-shaped IONPs demonstrate higher transverse relaxivity values [65,144,145,146,147,148]. Furthermore, zinc- and manganese-doped IONPs with high heating power have been shown to produce up to an eightfold increase in MRI contrast relative to pure IONPs [89,90,146,149,150]. Collective magnetic behavior in multicore IONPs, resulting in enhanced net magnetic moments, not only improves heating efficiency but also increases transverse relaxivity [69,151,152]. With respect to surface coating, long-chain polymers, thick silica shells, or hydrophobic polymers may reduce transverse relaxivity, as contrast strongly depends on the diffusion of water molecules near the magnetic core [153,154,155]. However, several studies indicate that coatings such as PEG, dendrons, and pH-sensitive hydrogels can enhance transverse relaxivity [89,143,156,157,158].

The MNPs being developed for MH are also explored via a novel emerging technique called Magnetic Particle Imaging (MPI) [159,160,161,162]. MPI exploits the nonlinear magnetization response of MNPs near zero magnetic field. In a standard MPI scanner, the static magnetic field is designed in such a way to contain a single zero-field region, called the field-free point (FFP). When an AMF is applied, only MNPs at the FFP contribute to the signal. Scanning the FFP across space enables reconstruction of the NPs distribution. MPI presents several major advantages over MRI: 1. It offers higher sensitivity than MRI, primarily determined by the magnetic moment of MNPs, which depends on M_s_ and crystal volume [163,164]. 2. It enables the use of multiple MNP classes with distinct magnetic responses, allowing separate image collection and color assignment to generate multi-color images. This permits in vivo studies of interactions between different cells loaded with specific MNP subclasses [165]. 3. The MPI signal is proportional to MNP concentration and is unaffected by NP aggregation, representing a major advantage over MRI. 4. It enables verification of MNP distribution at the tumor site before applying MH, a key advantage in systemic administration. While MPI AC fields at 20 kHz cause no tissue heating, increasing the frequency to 340 kHz induces detectable heating. Combined with gradient fields, heating is confined to the FFP, allowing selective tumor targeting without damaging surrounding tissues by positioning the FFP at tumor site [166]. 5. it lacks of background signal coming from the tissue. Although, MPI is limited by its lack of anatomical detail, requiring combination with a complementary morphological imaging modality, current efforts are directed toward developing a human scanner [167]. As MPI is in the early stages of biomedical application, ongoing technological advances are expected to broaden its medical potential [168].

## 3. Toxicity Issue, Biocompatibility, and Strategies to Improve Biocompatibility of Magnetic Nanomaterials

### 3.1. Toxicity Issues

Despite the promising biomedical potential of IONPs, they are not inherently risk-free. Concerns regarding both acute and chronic toxicity have raised, particularly under MH conditions, where deliberate heating of MNPs can inadvertently damage surrounding healthy tissues [169,170,171].

In vitro cytotoxicity studies have revealed that Fe_3_O_4_ NP-induced toxicity is both dose- and time-dependent, typically associated with increased production of reactive oxygen species (ROS) and lipid peroxidation, evidenced by elevated malondialdehyde (MDA) levels. This oxidative stress can impair enzymatic activity, damage membranes, and compromise cell viability. For instance, Ahamed et al. [172] reported significant ROS and MDA elevation in A431 and A549 cell lines exposed to 25–100 µg/mL MNPs, correlating with reduced cell viability. Similarly, studies on human umbilical vein endothelial cells (HUVECs) revealed oxidative stress and genotoxic effects at an IC_50_ of approximately 79 µg/mL MNPs [173]. Iron overload from internalized IONPs may further exacerbate toxicity by inducing ferroptosis—a form of programmed cell death driven by lipid peroxidation—and catalyzing Fenton-type reactions that generate hydroxyl radicals. These highly reactive species can cause DNA strand breaks, protein carbonylation, and membrane disruption. Such mechanisms have been implicated in neuropathological contexts such as intracerebral hemorrhage and may also contribute to nanotoxicity in non-neuronal tissues [174,175]. The surface chemistry of Fe_3_O_4_ plays a pivotal role in modulating their biocompatibility. For instance, in porcine aortic endothelial cells, dextran- or PEG-coated IONPs (5 nm and 30 nm) induced no significant loss of viability or ROS increase, even at 0.5 mg/mL after 24 h exposure. Notably, PEG reduced ROS production by 62.6%, and dextran by 35.2% compared to uncoated cores, with apoptosis levels remaining below 10% [176]. In contrast, polyethylenimine (PEI)-functionalized Fe_3_O_4_ NPs (30 nm) displayed pronounced cytotoxicity in SH-SY5Y, MCF-7, and U937 cell lines, decreasing viability by up to 50% within 24 h at 100 µg/mL, and even further after 168 h. These effects, characterized by increased ROS, lipid peroxidation, and lactate dehydrogenase (LDH) release, were largely mitigated upon PEGylation of the PEI coating [177].

In vivo, the toxicity profile of MNPs is influenced by parameters such as dose, administration route, biodistribution, and NP size. Following systemic administration, MNPs tend to accumulate in organs of the mononuclear phagocyte system, particularly the liver and spleen, where they may trigger inflammation and tissue injury. For instance, high-dose administration of dextran-coated IONPs in mice, followed by AMF exposure, resulted in severe hepatosplenic damage or mortality, whereas lower-dose groups survived but still exhibited tissue stress, including elevated liver enzymes and splenic necrosis [178]. NP size is equally critical: ultrasmall MNPs (2.3 and 4.2 nm) administered intravenously at 100 mg/kg induced fatal multiorgan oxidative stress, especially in cardiac tissue, whereas 9.3 nm particles of identical composition showed no overt toxicity at the same dose [179]. Long-term studies indicate that MNPs are only partially cleared from the body. Residual iron, often sequestered in ferritin-like structures, can persist in the liver and spleen for several months after administration. This persistence has been associated with chronic low-grade hepatic inflammation and disturbances in iron metabolism, although conclusive evidence linking this persistence to long-term health consequences remains limited [180,181].

### 3.2. Organic Coating

Extensive research was conducted on improving biocompatibility and cellular uptake through synthetic polymer coatings, such as PEG, polyvinylpyrrolidone (PVP), polydopamine (PDA), and PDA analogues, as well as naturally derived coatings like chitosan and dextran (Figure 1).

#### 3.2.1. Synthetic Polymers

PEG is one of the most widely used NP formulations, with broad applications due to its ability to reduce clearance and enhance water solubility. PEG forms a “stealth” hydration layer that decreases opsonization and uptake of mononuclear phagocyte system (MPS), while preventing hydrophobic NP aggregation and shielding the NP surface from enzymatic and antibody recognition [182,183,184,185]. PVP is a colorless, water-soluble, biocompatible polymer known for its exceptional pH stability and binding capabilities, which facilitate drug solubility and dispersion. Its amphiphilic structure enables effective interactions with solvents of varying polarities, making it highly versatile for constructing complex macromolecules, often through conjugation with polyacids such as polyvinyl alcohol (PVA) or polyacrylic acid (PAA). PVA-PVP composites further improve mechanical strength and thermal stability [186,187].

Polymerization of catecholamines leads to the formation of PDA and PDA-analogue polymers on the surface of NPs, particularly nanoclusters, providing multiple surface functions and enabling diverse applications. Dopamine and L-DOPA, members of the catecholamine class, can act as surfactants and are therefore suitable for one-step synthesis of core–shell structures via solvothermal/hydrothermal method. Magnetic nanoclusters (MNCs) containing a magnetite core and a polymeric shell synthesized in situ by a solvothermal process, using 3,4-dihydroxybenzhydrazide (DHBH) and poly [3,4-dihydroxybenzhydrazide] (PDHBH) as stabilizers, showed biocompatibility, antitumor efficacy and tumor selectivity against colon cancer cells (CACO2) and melanoma cells (A375) when applied in MH in vitro [188]. Other MNCs synthetized by solvothermal polyol reaction and coated with dopamine, 3,4-dihydroxybenzylamine, 2-aminomethyl-3-(3,4-dihydroxyphenyl) propionamide, and 3,4-dihydroxybenzylidenehydrazine yielded PDA and PDA-analogue coatings onto the core–shell structures. These NPs exhibited good biocompatibility in normal cells (fibroblasts and endothelial cells) as well as in melanoma cells (A375), and emitted a fluorescent signal, suitable for tumor imaging purposes [189].

#### 3.2.2. Natural Polymers

Chitosan, obtained through the deacetylation of chitin (present in insect exoskeletons), contains multiple hydroxyl (–OH) and amino (–NH_2_) functional groups that enable the binding of antitumor drugs such as paclitaxel (PTX). Chitosan is biocompatible, biodegradable, and exhibit antibacterial activity, making it a strong candidate for nanoscale drug delivery systems. Fe_3_O_4_ NPs coated with a chitosan shell have shown an 18% increase in paclitaxel loading compared to uncoated particles [190,191]. In addition, chitosan exhibits stimuli-responsive release behavior due to its pH-dependent solubility. However, while its mucoadhesive properties are advantageous for mucosal delivery, they may also reduce specificity by promoting of off-target accumulation in healthy tissues [192].

Dextran-coated IONPs are widely used due to their exceptional biocompatibility and enhanced magnetic performance. The dextran coating provides a stabilizing effect, preventing aggregation and preserving SP behavior. Moreover, dextran-coated IONPs have been successfully employed in drug delivery applications, demonstrating notable effects through controlled drug release [193,194].

## 4. Combination of Magnetic Hyperthermia with Other Therapeutic Modalities

### 4.1. Combination with Other Therapeutic Methods

Although MH can induce cell death on its own, it is particularly effective by sensitizing tumors to other treatments, demonstrating significant benefits as an adjunct therapy. For instance, studies have shown that MH can increase the proportion of complete responders to radiation therapy by 20 percentage points or more in breast, cervical, and head and neck cancers [195]. Similarly, a 2010 phase III trial in high-risk soft-tissue sarcoma showed that the addition of regional hyperthermia to chemotherapy nearly doubled the response rate (28.8% vs. 12.7% with chemotherapy alone; *p* = 0.002) [196].

Combining MH with modalities such as chemotherapy, radiotherapy, immunotherapy, and advanced drug carriers or natural compounds has shown synergistic efficacy in preclinical studies and emerging clinical applications [197]. IONPs remain a primary focus owing to their biocompatibility and strong heat induction; however, similar combination strategies are also being explored with other NP types, such as gold nanostructures for photothermal therapy and high-Z radiosensitizers [198,199].

**Figure 2:**
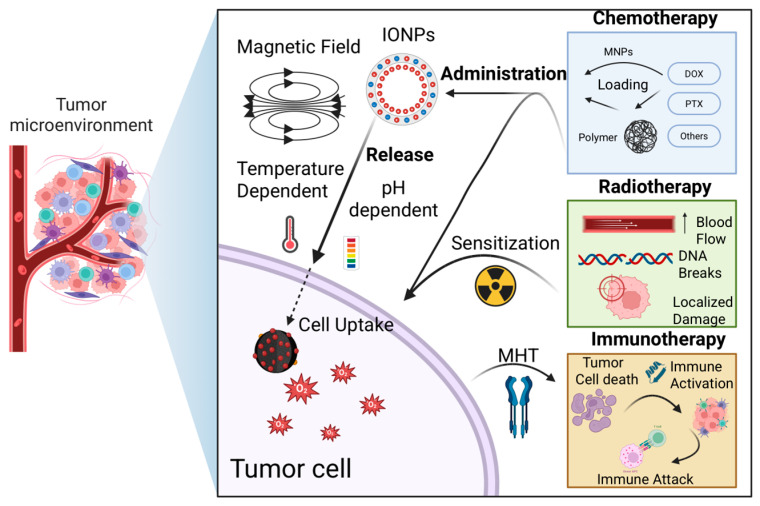
Chemotherapy—Nanoparticles can be loaded with chemotherapeutic agents such as doxorubicin, paclitaxel, gemcitabine, sorafenib, and platinum-based drugs, either by attaching them to surface polymers or directly incorporating them into the nanoparticles. Once internalized by tumor cells, the drug is more selectively released, a process facilitated by the hyperthermic effect or the acidic tumor microenvironment. This approach can help overcome multidrug resistance by enhancing drug uptake and efficacy. Radiotherapy—Radiation therapy increases local blood flow, thereby improving nanoparticle delivery to the tumor and enhancing tumor specificity. Additionally, irradiation induces DNA damage in tumor cells, making them more susceptible to hyperthermia-induced cell death when combined with MH. Immunotherapy—MH combination with immune-based therapies, such as interferons, interleukins, or PD-L1 immune checkpoint inhibitors can produce a synergistic effect. Destruction of tumor cells by MH releases tumor antigens, which, in conjunction with immunotherapy, stimulates both local and systemic immune responses against the tumor, potentially improving therapeutic outcomes.

Although both combination therapies effectively induce the death of tumor cells and stromal components, they carry the risk of selecting resistant tumor cell subpopulations [200], such as anastatic cells [201], blebbishield emergency program cells [202], phoenix rising cells [203,204], CASP3+ cell islands [205], nuclear expulsion cells [206], and cells undergoing senescence reversal [207], all of which are capable of repopulating the tumor. Nevertheless, due to their multi-targeted approach, combination therapies are generally considered less likely to promote emergence of resistant clones. Among current strategies, the most promising advance in oncotherapy is immunotherapy, which activates the body’s own immune system against tumor antigens released by the MH or other treatments. Such approaches have the potential to detect and eliminate distant and dormant tumor cells, ultimately establishing a durable antitumor immunity (Figure 2).

#### 4.1.1. Chemotherapy

**Figure 3:**
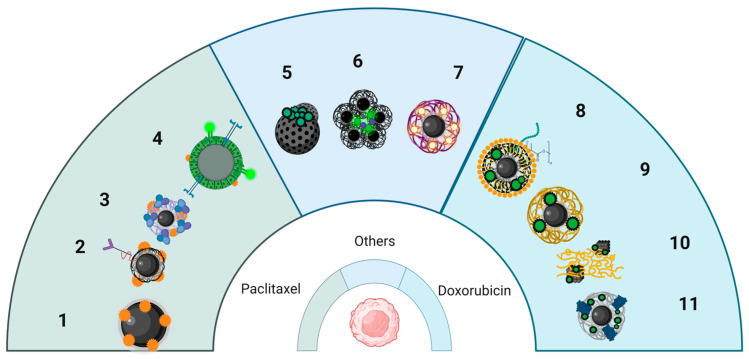
Schematic representation of several core–shell nanoplatforms, containing chemotherapeutic drugs. The nanoparticles with a IONP core and various shells were loaded with paclitaxel (left), doxorubicin (right) or others (such as cucurbituril, platinum-based prodrug, gemcitabine) used for tumor targeting, and combined MH with chemotherapy on in vitro/in vivo experimental models. Key: 1 = PTX bonded IONPs; 2 = SPIONs with PTX-loaded Chitosan; PEG and Folate ligand; 3 = SPIONs coated with lauric acid and human serum albumin as carriers for PTX; 4 = Mesoporous silica SPIONs with fluorescent folate ligand with CPT and PTX; 5 = MNP with nanovalves loaded with cucurbituril; 6 = Nanoflowers with ICG and Pt (IV) prodrug; 7 = SPIONs-PLGA with chitosan shell with gemcitabine; 8 = A54 peptide-functionalized PLGA micelles with DOX/SPIONs; 9 = DOX loaded IONPs with EDT coating; 10 = Fibers co-loaded with IONPs loaded with DOX; 11 = PEG-coated IONPs with LHRH containing DOX.

Moderate hyperthermia enhances tumor blood flow and cell membrane permeability, thereby improving drug delivery and facilitating drug uptake [208]. In addition, heating can interfere with DNA repair mechanisms and induce apoptosis, increasing the sensitivity of cancer cells to chemotherapeutic agents. In practice, IONPs have been engineered as dual hyperthermia and drug-delivery agents: they can be loaded or coated with chemotherapeutics (such as doxorubicin (DOX) or PTX) and subsequently heated remotely to simultaneously achieve drug release and cancer cells damage. For instance, in a murine breast cancer xenograft model, intratumoral injection of DOX-conjugated IONPs followed by exposure to AMF resulted in greater tumor regression than either MH or DOX alone, demonstrating a synergistic effect [209]. Similarly, PTX-loaded MNPs have shown synergistic efficacy with MH: one study reported both in vitro and in vivo evidence that PTX-bearing IONPs under AMF induced significantly higher cancer cell death and tumor growth inhibition compared to PTX or hyperthermia alone [210]. This combined approach can reduce the required drug dose (mitigating systemic side effects) while achieving enhanced tumor response (Figure 3).

Thermosensitive carriers can further enhance the synergy between chemotherapy and hyperthermia. For instance, magneto-liposomes or polymer-encapsulated IONPs can be engineered to release their payload when the local temperature rises during MH. In one approach, thermosensitive magnetic liposomes loaded with DOX and a cell-penetrating peptide achieved targeted drug release upon heating, significantly improving therapeutic outcomes both in vitro and in an MCF-7 xenograft murine model [211]. Thus, IONPs present a promising strategy for thermo-chemotherapy, offering noninvasive control and deep tissue penetration of the activating magnetic field, making them especially suitable for treating hard-to-reach tumors [30].

#### 4.1.2. Radiotherapy

Heat can radiosensitize tumor cells through several mechanisms. Hyperthermia induces protein and DNA damage and can inhibit the repair of radiation-induced DNA breaks, thereby increasing radiation efficacy. It also improves tumor oxygenation by increasing blood flow at mild temperatures, counteracting hypoxia-driven radio resistance, until higher temperatures may lead to vascular collapse and direct cell killing [195]. Moreover, while conventional hyperthermia techniques have struggled to achieve uniform heating of deep or irregularly shaped tumors [12], MH using IONPs offers a more targeted approach. IONPs can be delivered into the tumor either systemically or via direct injection and subsequently heated in situ by an external AMF, concentrating thermal damage within the tumor while sparing surrounding healthy tissue [195].

In human glioblastoma xenograft models, for instance, adjuvant MH elevated tumor cell DNA damage (γ-H2AX levels) and apoptosis when combined with radiation, translating to delayed tumor growth and prolonged survival in treated animals [208]. These benefits are currently being explored in clinical settings. In a single-arm pilot clinical study, intratumoral MH plus radiotherapy was evaluated in patients with recurrent glioblastoma multiforme. IONPs (aminosilane-coated Fe_3_O_4_) were injected into the tumor, and an AMF was applied to induce heating alongside fractionated radiotherapy. The combined treatment was found to be safe and led to prolonged survival compared to historical controls (median overall survival ~13–14 months after recurrence, significantly longer than with radiotherapy alone) [212]. This approach has since received regulatory approval in Europe as an adjunct therapy for glioblastoma, supporting the clinical potential of Fe_3_O_4_ NPs [213].

#### 4.1.3. Immunotherapy

MH can trigger immunogenic cell death (ICD) through the release of DAMPs such as ATP, HMGB1, and calreticulin that activate dendritic and T cells, thereby turning dying tumor cells into immunostimulants. Intracellular heating by IONPs induced broader ICD marker expression than external heating, highlighting MH’s unique immunological effect [214]. Thus, MH engages mechanisms that, beyond local tumor control, provide a pathway to systemic immune activation. Recent reviews highlight that NPs-mediated hyperthermia boosts tumor immunogenicity and permeability, promoting immune cell infiltration and increases responsiveness to checkpoint inhibitors [214,215]. In one model, MH combined with anti-CTLA-4 therapy suppressed both primary and metastatic tumors and induce long-term immune memory [216]. Other studies have showed that iron in SPIONs can shift macrophages to a tumor-suppressive phenotype [217]. Combining MH with immunotherapy may also reduce immune escape by enhancing antigen presentation and inducing inflammation within the tumor microenvironment [218,219]. Although still preclinical, these findings suggest MH may help turn immunologically “cold” tumors into “hot” responsive ones.

#### 4.1.4. Role of Natural Compounds and Polymer-Based Carriers in MH

Polymer and natural compound-based carriers enhance the functionality of IONPs by improving stability, enabling targeted delivery, and providing stimuli-responsive payload release. For instance, quercetin-loaded chitosan-coated MNPs demonstrated enhanced stability and tumor targeting in colon cancer models [220]. Similarly, polysaccharide-based magnetic hydrogels—such as chitosan-alginate combined with PNIPAM—achieved efficient on-demand drug delivery with controlled release under AMF heating [221]. These systems improved therapeutic specificity while reducing systemic toxicity, making them promising platforms for combined application of hyperthermia and pharmacotherapy.

Polymer coatings like chitosan and PEG enhance the stability, circulation, and drug loading capacity of IONPs. For instance, chitosan-coated IONPs, achieved a loading of ~3.2 mg_DOX_/mg_NPs_—six times higher than uncoated counterparts—and induced greater in vitro cancer cell death [222]. PEGylated Fe_e_O_4_ functionalized with folate or peptide ligands improved tumor uptake and PEG-related stability [223]. Such systems can also reduce premature clearance and minimize systemic toxicity.

Natural compounds may also act synergistically with MH. For instance, a PLA–PEG–curcumin–Fe_3_O_4_ formulation enabled AMF-triggered curcumin release and tumor shrinkage in vivo, outperforming curcumin or hyperthermia alone [224]. Other natural agents (e.g., resveratrol) and biodegradable hydrogels further enhanced MH’s versatility by providing controlled release and site-specific retention [221,225,226,227]. These strategies add multifunctionality while preserving biocompatibility.

Another strategy for designing MNPs involves targeting specific receptors on the cellular membrane, thereby providing highly specific therapeutic benefits. Using an engineered antibody fragment, Christian Ndong et al. successfully targeted folate receptor alpha-overexpressing cancer cells, resulting in high intracellular accumulation [228]. A similar formulation was developed by other group, achieving drug delivery, MRI, fluorescence imaging and cell targeting within the same formulation [229]. MNP formulations targeting the transferrin receptor have also been explored [230,231].

### 4.2. Experimental Studies of Biocompatibility and Oncologic Efficiency of IONPs In Vitro

#### 4.2.1. In Vitro Cancer Models Used for Testing of IONPs

Modern in vitro models are increasingly used to explore novel strategies for cancer detection and treatment, including the application of IONPs. These models range from traditional 2D cell cultures, where cancer cells are grown in monolayers, to more advanced 3D systems such as tumor spheroids and organoids, which more accurately replicate the tumor microenvironment. In addition, more complex simulated models are also being employed (Table 1).

Several 2D models have been widely adopted, yielding promising, reproducible, and accurate results. Accordingly, cancer cell lines, including human glioblastoma [232], lung [233], breast [234], cervical [235], pancreatic [229], and hepatic cancer [236], as well as murine breast/colon carcinoma [237] and fibrosarcoma [238], have been employed to evaluate MNP cytotoxicity, cellular uptake, and drug delivery efficacy. Fibroblast-like cell lines from humans [232] and mice [238,239] have also been used, primarily as controls. In addition, bacteria strains such as *Staphylococcus aureus*, *Proteus vulgaris*, and *Pseudomonas aeruginosa* have served as models for assessing the antibacterial activity of MNPs [233].

Three-dimensional tumor models are generally regarded as more accurate than monolayer-based systems for replicating tumor physiology and predicting responses to chemotherapeutic agents. A study conducted on breast cancer spheroids revealed that MCF-7 spheroids exhibited considerable heterogeneity, including marked differences in morphology. This variability indicates that such spheroids may not be optimal for evaluating anticancer drug toxicity or resistance [234]. In another study, porcine aortic endothelial cells (PAECs) were exposed to SPIONs to assess ROS production, cytoskeletal organization, and cell stiffness, with results showing significant and reproducible effects [240].

Norouzi et al. developed an MDCK-MDR1-GBM co-culture model to replicate the human blood–brain barrier (BBB) and glioblastoma (GBM) tumor interface. In this system, the MDCK-MDR1 layer, composed of kidney epithelial cells genetically engineered to overexpress the human MDR1 gene, served as BBB, while the GBM layer, consisting of human GMB U251 cells, was employed to evaluate MNP uptake and cytotoxicity [241].

#### 4.2.2. MNP Formulation

The primary type of iron-based NPs used in cancer therapy are IONPs, known for their magnetic properties and biocompatibility. Among them, SPIONs are particularly important, as they enable magnetic drug targeting, MH, and serve as contrast agents in MRI [229]. Research on iron-based NPs in cancer therapy generally follows two main approaches: (1) functionalized bare nanoparticles, evaluated for their biocompatibility, tumor-targeting capacity, and cytotoxic effects with and without MH, and (2) drug-loaded NPs, designed for delivering chemotherapeutic agents, photosensitizers, or to enable combined MH and chemotherapy.

Natural antioxidant-rich compounds play a dual role in nanoparticle synthesis: they act as reducing agents that promote NPs formation while preventing aggregation, and they serve as coatings that enhance the biocompatibility of MNPs by forming a protective antioxidant shell. Moreover, these compounds support the targeted delivery of bioactive agents into tumor cells. Many cancer cells are particularly sensitive to natural extracts containing polyphenols, resveratrol, flavonoids, or anthocyanins [242], which can trigger apoptosis or increase responsiveness to other treatments, including chemotherapy, photodynamic therapy (PDT), and hyperthermia. Natural compounds, such as polyphenols, can also act synergistically with anticancer drugs, including cisplatin, DOX, and 5-fluorouracil [243]. Rosemary terpenes have demonstrated antitumoral activity on colon cancer cells in vitro by inducing necrosis through an acute increase in ROS, and in in vivo colon cancer models, they inhibit proliferation and improve animal survival [244]. Green iron NPs (Rosemary-FeNPs), phyto-synthesized using the natural antioxidants from rosemary extract, exhibited an average diameter range of 50–100 nm with excellent homogenization [237]. Turmeric extracts and their key compounds, carnosic acid and curcumin, also showed antiproliferative effects of cancer cells [245]. SPIONs loaded with curcumin and coated with organic polymers, poly (lactic-co-glycolic acid)-poly (ethyleneglycol) di-block copolymer (PLGA-b-PEG) conjugated with the glycine–arginine–glycine–aspartic acid–serine (GRGDS) demonstrated targeted delivery. GRGDS peptide enables integrins targeting, which is typically overexpressed in cancer cells. Moreover, the combined delivery of curcumin enhanced therapy efficiency and offers a potential drug delivery platform for a chemotherapeutic, allowing a synergistic effect [232]. Several chemotherapeutic drugs, such as PTX [229,246], DOX [236,241,247], camptothecin (CPT) [229], gemcitabine [248], sorafenib [249], and temozolomide [208], have been loaded on MNPs. Although PTX is an effective antitumor agent, its clinical administration is challenging due to its hydrophobic nature. To address this limitation, MNPs loaded with PTX were synthesized using chitosan as coating: Fe_3_O_4_@LaF_3_:Ce^3+^,Tb^3+^/Chi NPs associated with PTX. This formulation increased PTX water solubility while preserving the SP properties of the MNPs, and provided a biocompatible method for PTX administration with reduced side-effects, enabling potential synergistic therapy combining MH and chemotherapy (Table 2) [246].

Folic acid receptors are overexpressed in many cancers; therefore, folic acid can be used for selective targeting of malignant cells in cancers including ovary, kidney, uterus, testis, brain, colon, lung, and myelocytic blood cells [250]. This strategy has been applied in the synthesis of PTX-loaded NPs, such as SPIONs functionalized with chitosan, PEG, and folic acid (FA), which demonstrated enhanced tumor targeting and increased PTX uptake in malignant cells [238]. In a study using SPIONs functionalized with a PLGA core and a poly(N-isopropylacrylamide)-carboxymethyl chitosan shell loaded with NU7441 and gemcitabine, targeting folate receptors increased specific cellular uptake, while the pH-sensitive shell facilitated preferential gemcitabine release in the tumor environment. The nanoplatform retained magnetic properties, making it suitable for combined MH and chemotherapy [248]. Another approach utilized coatings such as lauric acid (LA) and human serum albumin (HSA) for SPIONs (SPION-LA-HSA-Ptx) to delivery PTX. LA improved the loading of the hydrophobic drug PTX and NPs stability, while LA and HSA enhanced the biocompatibility and colloidal stability of the MNPs [234]. MNPs coated with an amphiphilic polymer containing disulfide linkages (hyaluronic acid–disulfide bond–polylactic acid) and loaded with PTX demonstrated efficient drug delivery by combining magnetic tumor targeting and redox-triggered specific release of PTX, resulting in improved therapeutic efficacy and minimized side effects [235]. Additionally, mesoporous silica-coated SPIONs with fluorescent dyes, hydrophilic groups, cancer-specific targeting ligands, and co-loaded with CPT and PTX offered advantages in magnetic manipulation, targeted drug delivery, and efficient drug loading and release [229].

A54-Dex-PLGA micelles loaded with DOX exhibited high encapsulation efficiency (approximately 80%) and sustained release for up to 72 h. MNPs demonstrated tumor targeting and enhanced efficacy compared to free drug [236]. DOX-loaded IONPs with surface coatings, such as trimethoxysilylpropyl-ethylenediamine triacetic acid (EDT), were also effective, as the EDT coating significantly enhanced blood–brain barrier penetration. Moreover, this formulation provided sustained DOX release, with faster release under acidic conditions (tumor microenvironment), allowing more tailored therapeutic action [241]. The DOX-loaded Fe_3_O_4_@MnO_2_@PPy nanocomposite improved hypoxia tolerance and PDT efficiency by integrating photothermal, photodynamic, and chemotherapeutic treatments [247].

SPIONs synthesized using a double coating of PVA or PEG and magnesium–aluminum-layered double hydroxide (MLDH) were loaded with sorafenib. The resulting NPs were spherical, with an average diameter of 17 nm, and released sorafenib over a period of 72 h, with enhanced release under acidic conditions (pH 4.8) simulating the tumor microenvironment. This system exhibited increased toxicity towards HepG2 hepatoma cells and reduced toxicity towards fibroblast 3T3 cells, used as controls, compared to free sorafenib [249].

#### 4.2.3. Efficiency, Side Effects

IONPs have been extensively studied and applied in cancer therapy due to their effectiveness in targeted treatment, imaging, and hyperthermia, along with their generally favorable biocompatibility.

The cytotoxic effects of IONPs may result either from hyperthermia-induced mechanisms [232] or from the intrinsic properties of the nanoparticles themselves [233]. Functionalization has been shown to enhance both cytotoxicity against cancer cells and intracellular targeting. For instance, MNPs incorporating rosemary flavonoid compounds demonstrated improved efficacy [237]. Tran et al. reported that coating IONPS with chitosan or PVA reduced toxicity towards normal mouse fibroblasts, with PVA showing superior results and also decreasing NP aggregation, highlighting the crucial role of surface coatings in biocompatibility [251].

The generation of ROS has been identified as a key mechanism driving the biological activity and toxicity of NPs, contributing not only to their antibacterial effects [233] but also to cellular morphological changes and the formation of actin stress fibers [240]. Overall, the use of IONPs loaded with various anticancer agents has shown promise, enhanced therapeutic efficacy and targeting specificity while minimizing adverse side effects. MNPs loaded with PTX demonstrated increased tumor cell toxicity compared to free PTX, with reduced side effects and improved imaging capabilities via both MRI and fluorescence imaging [246]. These NPs also induced apoptosis in cancer cells [238] and revealed high potential of magnetically guided targeted drug delivery in breast cancer, although some studies reported effects comparable to PTX alone [234]. Furthermore, PTX-loaded MNPs have been employed for targeted administration that combines magnetic drug delivery with redox dependent release, thereby enhancing cytotoxicity [235].

DOX-loaded IONPs have shown considerable potential for cancer treatment. DOX-loaded IONPs with surface coatings such as EDT were able to overcome multidrug-resistant cancer cells (MDR) in a GBM model, achieving magnetic targeting with low systemic toxicity [241]. Popescu et al. demonstrated the direct delivery of DOX-loaded IONPs into the cytoplasm via macropinocytosis and endocytosis, highlighting promising future possibilities [252]. Additionally, Fe_3_O_4_@MnO_2_@PPy nanocomposites employed DOX-loaded IONPs to deliver the chemotherapeutic agent and enhance both PDT and PTTat the tumor site, resulting in more effective cancer treatment [247].

#### 4.2.4. Type of Cell Death

- Apoptosis

**Figure 4:**
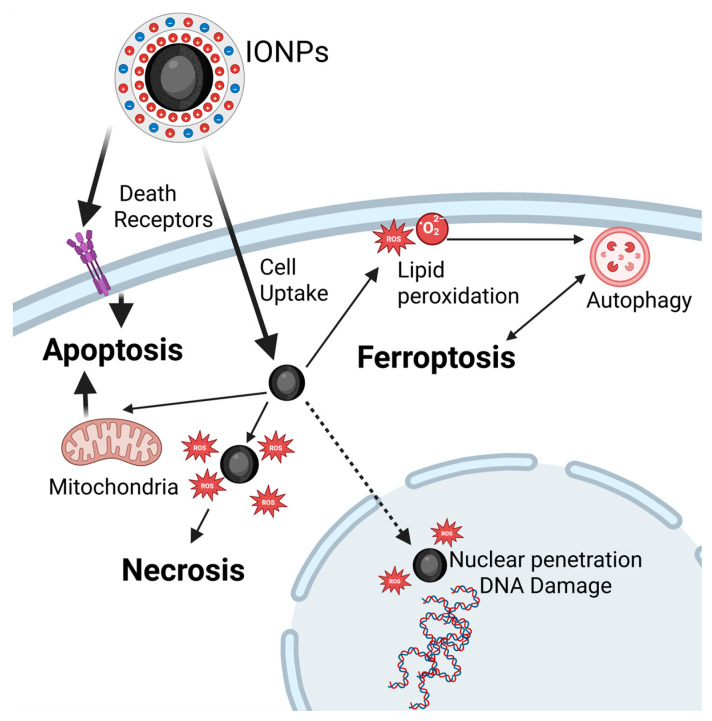
Mechanisms of cell death induced by MH. Following cellular uptake, and exposure to AMF, MH triggers apoptosis via either the membrane pathway, through death receptor activation, or the mitochondrial pathway, both leading to caspase cascade activation. Higher temperatures, nutrient deprivation, or combined therapies favor necrosis, typically caused by rapid ROS accumulation that overwhelms antioxidant defenses, leading to terminal oxidation of cellular components. ROS can also damage nuclear DNA, leading to necroptosis or activating DNA repair. Ferroptosis results from intracellular iron buildup, which generates ROS through Fenton reactions, and causes lipid peroxidation. This process can culminate in cell death or trigger autophagy, particularly described on in vivo models, with lysosomal activation. Autophagy may enable cell survival by recycling damaged organelles to provide energy and restore cellular functions acting as a tumor escape mechanism. However, when damage is extensive, autophagy serves as a programmed cell death pathway.

NPs, depending on their dose and physicochemical properties, can influence various cell fates (Figure 4), including necrosis and apoptosis [253]. Apoptosis is a distinct form of cell death characterized by specific morphological changes, such as membrane blebbing, cell shrinkage, chromatin condensation, and the formation of small vesicles (apoptotic bodies) [254]. The key mechanism of apoptosis occurs via three main pathways involving death receptors, mitochondria, or the endoplasmic reticulum, with caspases mediating all morphological and biochemical changes [255,256]. Consequently, multiple studies highlight the importance of correlating NPs dose and exposure time with the extent of apoptosis. For instance, Gong et al. demonstrated that the proportion of apoptotic cells in human umbilical vein endothelial cells (HUVECs) exposed to SP core/shell MNPs was dependent on both NPs concentration and exposure duration. Specifically, a significant increase in apoptosis was observed after exposure to at least 25 μg/mL of 50 nm NPs for more than 12 hours, and at least 50 μg/mL of 30 nm NPs for a minimum of 24 hours [257]. Functionalized IONPs, particularly those conjugated with therapeutic agents—such as IONPs conjugated with lysine and methotrexate tested on breast cancer—can effectively induce apoptosis in vitro [258]. Similarly, Tousi et al. reported that mPEG-b-PLGA coated IONPs loaded with the flavonoid eupatorin increased apoptosis and reduced necrosis in prostate cancer cells compared with free eupatorin or uncoated IONPs, suggesting their potential as an effective drug delivery system for cancer therapy [259].

- Necrosis

Necrosis has long been considered the result of general cell injury caused by trauma. Consequently, it is viewed as an uncontrolled form of cell death that is not triggered by specific signaling pathways. Various clinical conditions, including toxin exposure, ischemia, and viral or bacterial infection can induce necrotic cell death [253]. In this context, ROS appear to play a key role, as demonstrated by Khan et al., who reported that ROS generated by IONPs induce necrosis and cell death in lung cancer cells (Figure 4). The type of cell death—necrosis vs. apoptosis—is determined by the level of oxidative stress and cell’s antioxidant capacity [260]. Another study showed that certain coatings and higher doses of IONPs, particularly PEI coatings known for their cytotoxicity and membrane-damaging effects, can induce necrosis [185]. Only a few in vitro studies have specifically addressed necrosis caused by IONPs (Figure 4). Most research focuses on apoptosis, with necrosis occurring as a secondary outcome at higher doses, often associated with increased oxidative stress or membrane damage [257,261].

- Ferroptosis

Ferroptosis represents a form of regulated cell death caused by to lethal intracellular accumulation of iron. It is primarily driven by the buildup of ROS, which lead to lipid peroxidation, membrane instability and ultimately cell death. One key pathway through which IONPs induce ferroptosis involves their intracellular accumulation and internalization into lysosomes, where they are degraded, resulting in the release of iron ions (Figure 4). Ferroptosis is driven by ferrous iron through Fenton reactions, generating ROS—including hydroxyl radical (HO·), which intensify lipid peroxidation [262]—disrupting mitochondria functions [263,264], causing membrane rupture [265], and inducing other ultrastructural alterations in organelles such as endoplasmic reticulum and peroxisomes [266,267]. Cellular antioxidant defenses, including the cysteine/glutamate antiporter and the FSP1/ubiquinol systems can be overwhelmed during ferroptosis. This pathway represents a promising target for current and future anticancer therapies, as tumor cells are particularly susceptible [268,269,270]. In vivo, ferroptosis has been considered a potential mechanism for tumors destruction; however, accumulation of IONPs in healthy tissues limits its therapeutic application. Drugs associated with ferroptosis, such as lanperisone, lorafenib, trigonelline, cisplatin, and ferumoxytol have been used to selectively target tumors [262]. IONPs are especially suited to induce ferroptosis via multiple mechanisms and may serve as theranostic agents, providing both therapeutic and diagnostic functions. Another approach involves synergistic therapies. For instance, Qi Nie et al. demonstrated that IONPs loaded with PTX increased intracellular iron concentration, enhanced ROS production and confirmed ferroptosis through upregulation of ferroptotic markers [271]. Similar findings were reported in studies targeting non-small cell lung cancer cells. Ferroptosis is also linked to autophagy, as several studies suggested [272,273]. Autophagy—the physiological process of degrading and recycling organelles—can protect against tumor development but may also contribute to cell death in healthy tissues. When stressors such as IONPs accumulate in healthy tissues, elevated ROS levels can modify the intracellular environment, causing autophagy to shift from a protective mechanism to a promoter of cancer progression [274].

### 4.3. Biocompatibility and Oncologic Efficiency of IONPs In Vivo

In vivo studies have been conducted for the theranostic applications of IONPs, SPIONs, surface-coated IONPs, charged PVA-coated SPIONs, protein-coated IONPs, SPIONs coated with anti-biofouling polymers, among others. Technological advances enable drug delivery at the nanoscale within tumors, with their small size and surface coatings allowing transport even through tumor stromal components.

#### 4.3.1. Biodistribution

**Figure 5:**
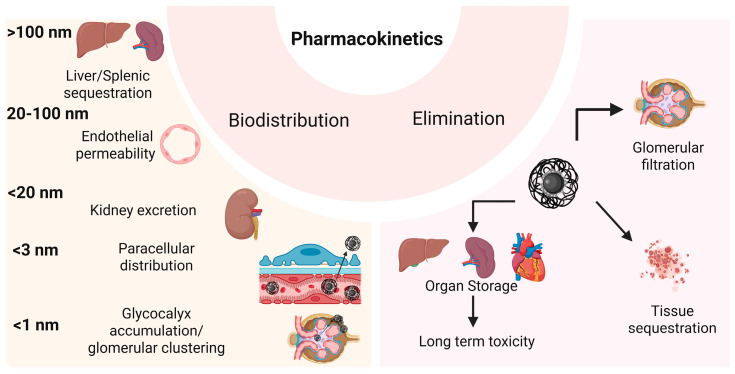
Pharmacokinetics of IONPs in vivo after systemic administration or vascular leakage post-intratumoral injection. Biodistribution of the MNPs strongly depends on their size, with nanoparticles between 20 and 100 nm diameter being the most suitable for MH, due to their ability to pass through the endothelial layer into the tissue leading to selective tumor accumulation (EPR effect), that can be enhanced by application of an external magnetic field gradient. Smaller particles (<1 nm) are fast eliminated by the kidney or can cluster into the glomerular cells glycocalyx leading to impaired nephron function, while bigger MNPs (>100 nm) can be stored in the internal organs, leading to medium/long term toxicity.

IONP biodistribution depends on several factors, including their size and shape as well as the type, chemical composition, and surface charges of their coating properties that also influence their ability to migrate into healthy tissues. In living organisms, IONPs may follow different pathways, particularly depending on their size (Figure 5). NPs with diameters greater than 100 nm are rapidly captured in the spleen and liver, and their penetration into tumors is limited by the pathological characteristics of the tumor vasculature, which vary with cancer type and stage [275]. Wang et al. reported that after oral administration of IONPs smaller than 100 nm in mice, the liver exhibited two picks in NP concentration: on the first day and seventh day. These NPs also accumulated in other organs, reaching maximum levels at 6 h post-gavage (lungs, kidneys), on the first day (stomach, small intestine, bone marrow), and during the first three days (heart, spleen, brain) [276].

IONPs with sizes between 20 nm and 100 nm are preferred for cancer therapy, to avoid rapid urinary excretion (<20 nm) and uptake by the spleen and liver (>100 nm) [277]. Several studies have highlighted their effects on vascular permeability. In tumors with poor vascularization, administration of IONPs combined with external AMF exposure induces alterations in the endothelial layer, enhancing drug accumulation within the tumor [278]. IONPs can also increase endothelial permeability by generating oxidative stress, which reorganizes microtubule positioning within the cellular cytoskeleton [279].

Small IONPs, with sizes below 20 nm, can readily cross the endothelium to reach various organs and may be filtered by the kidneys. These processes occur following intravenous or oral administration, or when NPs migrate from the tumor back into the bloodstream [280]. Since the endothelial glycocalyx contains gaps of approximately 20 nm between proteoglycan chains, small NPs can freely traverse the healthy endothelium layer [281]. In tumors, the endothelial layer develops pores ranging from 100 nm till 1 µm (with pore size depending on cancer type and stage), which can be easily penetrated by small IONPs (<20 nm) [282].

Injected IONPs with sizes below 10 nm are largely excreted via urine. Studies have shown that more than 40% of administered IONPs are eliminated within 24 h. PEGylated IONPs with sizes around 10 nm are readily transported into cells and accumulate at high concentrations in tumors, as well as in the spleen and liver, where their degradation occurs very slowly (Figure 5). At high concentrations, the PEGylated IONPs can be toxic and may induce autophagy [185].

IONPs smaller than 3 nm can also pass through vessel walls via the paracellular route [283], whereas NPs around 1 nm are retained in the glycocalyx of the glomerular filtration membrane, forming nanoclusters that persist in the kidney for extended periods [284].

#### 4.3.2. Coating

For drug delivery, IONPs are coated with various materials, including natural polymers (dextran, chitosan, starch, etc.) [285], synthetic polymers (PEG, PVA, PVP, etc.) [285,286], proteins (albumin) [287], lipids [288], silane [289], silica [290], and combinations of synergistic materials [291]. To prevent IONP agglomeration, which can cause embolism in capillaries [292], and to reduce rapid systemic dispersion, functional groups such as amines, aldehydes, thiols, and carboxylic acids are employed during IONP synthesis [293]. IONPs can be synthesized with specific coatings that either enhance the cytotoxicity of the transported drug such as gold-coated IONPs [294], or enable prolonged drug release, lasting up to four days post-administration, as seen with hyaluronic acid-coated IONPs [295]. Intraperitoneal administration of gold-coated IONPs in mice bearing melanoma tumor led to significant NPs accumulation in the tumor, as well as in spleen, liver, kidney, lungs and brain, with associated ultrastructural tissue modifications [296].

#### 4.3.3. Shape

IONPs have been synthesized in various shapes, including cubes, concave cubes, spheres, tetrahedrons, hexagons, octahedrons, octapods, polypods [297], ellipsoids, discs, cylinders, cones, and hemispheres [277]. The shape of IONPs play a critical role in their transport through the circulatory system and in drug delivery to target tissues. Elongated drug carriers travel closer to the vessel wall compared to spherical nanosystems, a margination property that facilitates the transport of these NPs into adjacent tissues [298]. Spike-shaped IONPs readily adhere to the endothelium, while elongated IONPs interact with the vessel wall along their long axis. These interactions can retain the NPs in regions unrelated to the treatment site, thereby limiting their delivery to the tumor when administered via intravenous or intra-arterial injections [299]. In contrast, spherical NPs are more efficiently transported through the circulatory system due to their smaller contact area [277].

#### 4.3.4. Electrical Charge

The zeta potential of IONPs significantly influences their cellular uptake. Several studies report higher internalization for IONPs with a positive zeta potential [300]. The penetration of IONPs into cells is affected by their size, the chemical composition of their coating, hydrophobicity, and the proteins that can adsorb onto their surface. Administration of positively charged IONPs (30 nm, 10 mg/kg, for 8 days) in pregnant mice resulted in fetal death and accumulation of NPs in the fetal liver and placenta [301]. Due to their hydrophilic properties, PEGylated IONPs exhibited prolonged systemic circulation, whereas PVP-coated IONPs demonstrated anti-opsonization properties [277]. The type of cell exposed to IONPs also plays a crucial role in determining NPs internalization [302]. Moreover, cancer tissues are more acidic than healthy tissues, a characteristic that has driven the development of IONPs capable of targeted drug attachment and pH-responsive release [303].

#### 4.3.5. IONPs Internalization

Inside the cell, IONPs can bind various molecules or ions, triggering reactions that may influence the intended outcome [304]. Spherical IONPs are captured by lysosomes, whereas spherical, elongated, or spiked IONPs are transported along the microtubules within endosomes to the storage and processing regions. Ultrasmall spherical IONPs are rapidly transported to the nucleus, where they inhibit DNA synthesis and induce apoptosis [305]. Similar DNA effects were observed in a study performed in mice following intravenous administration of ultrasmall IONPs (4–6 nm) [306]. Hexagonal-shaped NPs, in contrast, remain in the cytoplasm [277] where they induce oxidative stress.

#### 4.3.6. Immune Response Following IONPs Administration

Systemic administration of IONPs triggers complex responses of the organism. Delivery of IONPs via inhalations, injections (intraperitoneal, intravenous, intraarterial) or oral gavage has been shown to decrease immune responses by suppressing the activity of helper T lymphocytes [307,308]. Macrophages efficiently phagocytize large IONPs [309], IONPs with positive zeta potential [310], or those with a spherical shape. Elliptical NPs are phagocytized by macrophages in less than 6 min when the initial contact occurs with the major axis perpendicular to the phagocyte membrane [311], whereas other orientations can delay phagocytosis for hours [312]. Worm-shaped IONPs evade macrophage phagocytosis [313] and exhibit higher tumor accumulation compared to spherical NPs [314].

#### 4.3.7. Routes of Administration and Toxicity

Intratumoral injection of IONPs is considered the most efficient delivery method, as it avoids systemic responses; however, the potential for migration from tumor site into the circulatory system or surrounding tissues cannot be overlooked and requires further investigation. IONPs can be engineered with specific shapes, sizes, and chemical compositions to enable controlled drug delivery and release, and they can also be applied in MH to induce apoptosis in tumor cells. Wojtera et al. investigated the role of iron content in nanostructures exposed to AMF and demonstrated that a higher iron concentration generates large amount of heat [315].

The application of a magnetic field gradient to direct the IONPs toward tumor sites can be influenced by other electromagnetic fields (wi-fi, static magnetic fields, etc.) [316,317]. The behavior of IONPs depends not only on their size but also on their composition, as well as on the local organization and structure of IONPs assemblies formed under AMF [318]. Additionally, the number and arrangement of blood vessels inside the tumor can impact drug delivery [319]. Transport of IONPs through tissues using a magnetic field gradient may also interfere with the function of healthy organs and blood vessels. This risk can be minimized by using intratumoral administration. For instance, Johannsen et al. injected intratumorally 13 nm IONPs in rats with prostate cancer for MH (45 °C or 50 °C, for 30 min), resulting in homogenous distribution of NPs within the tumor; however, not all animals survived the treatment [320].

The toxicity of IONPs depends on several factors, including NPs properties, characteristics of healthy tissues, tumor specificity, and exposure to AMF [140]. Wu et al. highlighted the important role of IONP size in toxicity. Intravenous administration of 2.3 nm or of 4.2 nm IONPs at 100 mg/kg resulted the mouse mortality, likely due to cardiac failure, the results indicating increased oxidative stress in the heart, lungs, liver, spleen, and serum. In contrast, no mortality was observed when the same dose was administered using 9.3 nm IONPs [179]. Oral administration of 30 nm IONPs by gavage for 5 days in Wistar rats showed that 500 mg/kg induced anorexia and lethargy, while 5000 mg/kg cause severe effects such as ataxia, respiratory arrhythmia, pulmonary and cardiac hemorrhages, and liver degeneration [321]. A previous study reported hepatotoxic effects in Wistar rats following oral administration of a lower doze (150 µg/kg) of 30 nm IONPs for a longer duration (15 days) [322].

#### 4.3.8. Elimination

IONPs can be excreted from the body if certain conditions are met: their size, shape, zeta potential and other physicochemical properties must prevent dispersion, agglomeration and long-term storage, while allowing glomerular filtration. Following systemic administration, IONPs can accumulate in specific organs depending on both their intrinsic properties and the characteristics of the target tissues [323]. IONPs may interfere with iron metabolism, participate in electron transfer reactions, or interact with proteins; they are also immunogenic and can promote oxidative stress, thereby triggering ferroptosis. In vivo or in clinical trials have investigated IONPs for the treatment of various cancers, either through hyperthermia (induced by radiofrequencies, microwaves, magnetic field excitation, or ultrasounds) or in combination with radiotherapy.

#### 4.3.9. Combined Radiotherapy and MH

MH can be combined with radiotherapy, as the effects of radiotherapy are enhanced by heating the administered IONPs [195]. Several IONP formulations have been developed and investigated for radiosensitization, including dextran-coated IONPs (in prostate carcinoma and GBM), gold-coated IONPs (in melanoma), and sodium citrate-coating IONPs (in breast adenocarcinoma) [308]. Li et al. studied the effects of SP IONPs (60 nm), capable of pH-responsive self-assembly, in a mouse model of non-small cell lung cancer. The nanoclusters were administered intratracheally, followed by radiotherapy, and all treated animals survived with reduced tumor areas [324]. Zhu et al. examined the biodistribution of intratracheally administrated IONPs (22 nm) in Sprague-Dawley rats and observed their presence in the systemic circulatory system within 10 min after instillation, as well as long-term accumulation in the liver, kidney, and spleen up to 50 days post-administration [325]. Hyaluronic acid-based IONPs (40 mg/kg, peritumoral injections) were tested for radiosensitization in mice bearing subcutaneous tumors; when combined with X-ray irradiation, this treatment resulted in significant tumor reduction and 100% survival, as reported by Bae et al. [326]. Overall, the available literature provides conflicting results regarding the effects of IONPs on cancer progression.

## 5. Clinical Translation and Studies

The importance and relevance of the topic are highlighted by the large number and diversity of clinical studies conducted in recent years, and particularly by the fact that several IONPs have already been approved for clinical use. For instance, Nanotherm (MagForce Ag, Berlin, Germany), consisting of SPIONs, (Fe_3_O_4_ or γ-Fe_2_O_3_) with an aminosilane coating has been approved by both the EMA and FDA for use in MH therapy in recurrent GBM and prostate cancer [19,24]. Other FDA-approved NPs, although not applied in cancer therapy, include Ferumoxytol (Feraheme, SPIONs (Fe_3_O_4_) coated with polyglucose sorbitol carboxymethyl ether, produced by AMAG Pharmaceuticals, Inc., Cambridge, MA, USA), indicated for the treatment of iron deficiency anemia in patients with chronic kidney disease; Fermoxtran-10 (Sinerem, produced by Guerbet, Saint-Ouen, France, know as Combidex when produced by Advanced Magnetics, Cambridge, MA, USA), composed of SPIONs (Fe_3_O_4_) coated with dextran and developed as an MRI contrast agent; and Ferumoxsil (Lumirem- Guerbet), SPIONs (Fe_3_O_4_) formulation with a siloxane coating, used as an oral gastrointestinal tract imaging agent [19].

Clinical trials have investigated the diagnostic and therapeutic properties of IONPs in various malignancies. In a phase 1 clinical trial, Carbon Nanoparticle-Loaded Iron (CNSI-Fe(II)) was administrated at doses of 30, 60, 90, and 150 mg in patients with breast cancer and other advanced solid tumors. A partial response was observed in 25% of patients receiving 60 mg, while serious adverse events occurred in 33.3%, 25%, 83.3%, and 0% of patients at the 30, 60, 90, and 150 mg doses, respectively [327]. Regarding the diagnostic role of SPIONs, two clinical trials highlighted their advantages in sentinel lymph node detection. The use of MagTrace (SPIONs coated with carboxydextran; Sysmex Europe SE, Norderstedt, Germany) achieved a 100% detection rate with no reported adverse effects [328], and SPIONs identified more sentinel lymph nodes compared to radioisotopes (97.4% vs. 91.2%, *p* = 0.057) [329]. Additionally, NanoTherm represents the first nanoparticle-based therapy approved for the treatment of GBM multiforme. Several clinical trials have been conducted in patients with vascular impairments (such as coronary artery disease and cerebral ischemia), in those with impaired kidney function, and in various malignancies (including melanoma, carcinoma, and esophageal, rectal, breast, and prostate cancers) for the evaluation of lymph nodes. Ultrasmall SPIONs have also been applied in combination with diffusion-weighted magnetic resonance imaging (DW-MRI) in clinical trials to assess neoplastic changes in lymph nodes [330]. An ongoing clinical trial, ANCHIALE, is currently recruiting in Poland to further evaluate its efficacy and tolerability in glioblastoma patients [331].

Intra-venous injection of SPION-based contrast agent Ferumoxytol has proved effective in identifying liver neoplasms by enhancing liver heterogeneity in MRI scans, thereby enabling more accurate characterization of liver function and tumors [332].

In rectal cancer, clinical trials are currently investigating a NanoEcho Particle-1 (NEP-1, Ferumoxtran Lyophilisate 20 mg_Fe_/mL) based contrast agent to improve the diagnosis of lymph node metastases and staging [333]. Some of the major issues that need to be solved are represented by technical problems like precise NP delivery, uniform heating [24], and adverse local and general effects.

Combination therapy with MH and radiotherapy has also been evaluated in a clinical study involving 66 patients with GBM. Patients received intratumoral IONPs followed by MH and radiotherapy, during which adverse effects were reported, including elevated body temperature (38 °C), headaches (likely due to increased intracranial pressure), convulsions, motor impairments, tachycardia, and blood pressure fluctuations [212].

Adverse reactions reported in clinical trials have limited the use of IONPs in patients, including pain, hypotension, vasodilation, paresthesia [334], hypersensitivity, and anaphylactic shock [335]. Nanotherm has been shown to be an effective thermotherapy agent when combined with radiotherapy; however, its use has been associated with side effects such as fever, headaches, convulsions, and cardiovascular alterations. In advanced solid tumors, CNSI-Fe(II) demonstrated dose-dependent adverse effects and partial tumor responses at certain dosages, highlighting the need for careful dose optimization [327]. Ferumoxytol also faces specificity challenges due to the overlap between inflammatory and tumor signals [332]. NanoEcho’s ongoing work illustrates the time-consuming nature of clinical trial recruitment and regulatory approval processes [333]. Key lessons for future research include the recognition that iron oxide nanoparticles hold significant potential for theranostics, integrating both imaging and therapy [332]. Direct destruction of cancer tissue can be achieved through IONP-induced heating at temperatures above 45 °C; however, this approach may also damage healthy cells when the tumor is not well delineated [336,337]. Most clinical trials have employed IONPs for tumor or metastasis identification and demarcation [338], drug delivery [339], and enhancement of radiotherapy or chemotherapy [340]. Adjusting dosage to regulate thermal effects has improved both the safety and efficacy of therapy [327]. Moreover, ultra-low-dose SPIONs imaging provides an effective balance between detection sensitivity and minimization of side effects [328].

To optimize selective tumor accumulation, the application of an external AMF to IONPs can direct them toward the targeted tissue [341]. Magnetic guidance may enhance tumor accumulation and potentially reach metastatic regions that are often unresponsive to conventional treatments such as chemotherapy or radiotherapy. However, a major issue is the potential damage along the path of the NPs, as their accumulation may injure vessels and tissues, partly through the induction of indirect ferroptotic cell death. Intratumoral injection or direct administration of IONPs into tumor-feeding arteries is technically challenging and, in some metastatic sites, practically unfeasible. Therefore, the secondary effects of systemic IONP administration must be carefully considered in future studies, including kidney accumulation and clearance (which depend on the physicochemical properties of the NPs) [140], off-target tissue deposition, oxidative stress [261], and ferroptosis in healthy cells.

In terms of biocompatibility and tumor accumulation, surface coatings with aminosilane or dextran have been shown to improve the properties of NPs [19]. Overall, the physicochemical characteristics of NPs, such as size, shape, and surface charge, should minimize secondary effects. Coatings are therefore required to ensure biocompatibility and targeting capabilities without compromising magnetic properties, while also enabling selective tumor accumulation. Upon exposure to an AMF, these NPs should be capable of generating sufficient heat within the tissue to effectively destroy tumor cells.

Despite the encouraging results reported to date, many challenges remain, particularly regarding interactions with biological tissues. Key issues include optimizing the route of administration, enhancing tumor selectivity and accumulation to achieve effective tumor destruction without harming healthy tissue, and ensuring safe elimination from the body. Consequently, further research is required to address these limitations and advance MH toward clinical application as an adjuvant, palliative, or second-line therapy for solid tumors.

**Table 1:** Biocompatibility and anti-cancer efficacy of iron oxide nanoparticles with and without magnetic hyperthermia in vitro.

Nanoparticles	Model	Main Results
IONPs with PEG coating/IONPs with PEI coating	SKOV-3 human ovarian cancer/RAW 264.7 murine macrophages	Cytotoxic effects by ROS production and apoptosis induction [185]
SPIONs loaded with curcumin, coated with poly (lactic-co-glycolic acid)-poly (ethylene glycol) di-block copolymer (PLGA-b-PEG) conjugated with glycine-arginine-glycine-aspartic acid-serine (GRGDS)	T98G-glioblastoma multiforme, fibroblast-like cell line	Induced cytotoxic effects increased by exposure to radiofrequency hyperthermia application [231]
IONPs	A549 human lung cancer cell line Staphylococcus aureus, Proteus vulgaris, Pseudomonas aeruginosa	Cytotoxic effect Antibacterial effect through ROS generation [232]
SPIONS functionalized with SDS and loaded with curcumin and coated with chitosan SPIONs-SDS-CU-CHIT	HeLAa human cervical cancer	Decreased viability in a dose and time related manner related to drug release in the medium [234]
Green iron nanoparticles (Rosemary-FeNPs)	4T1 murine breast cancer C26 cancer cell lines	Cytotoxic effect against cancer cells, efficient intracellular delivery of the rosemary flavonoid components [236]
Bare superparamagnetic iron oxide nanoparticles (SPIONs)	Porcine aortic endothelial cells (PAEC)	ROS formation leads to morphological changes and forms actin stress fibers; blocking ROS formation by functionalization could increase medical applications [239]
IONPs coated with chitosan IONPs coated with polyvinyl alcohol (PVA)	Human fibroblasts	IONPs coated with chitosan induced mild toxicity, IONPs coated with PVA were well tolerated [240]
ferumoxytol carboxymethyldextran coating	mammary adeno carcinoma cells incubated with macrophages	Macrophages showed pro-inflammatory M1 phenotype upon ferumoxytol exposure Increased caspase -3 in mammary tumor cells [342]
IONPs loaded with LLY-507 (inhibitor of SMYD2), coated with PVA	A549 human non-small cell lung cancer cell line RBC- human	Efficient delivery of the SMYD2 inhibitor by the IONPs, dose dependent decrease in viability, hemolysis below 5% [343]
poly(ethylene glycol)-*block*-poly(lactic-*co*-glycolic acid) copolymer-encapsulated Fe_3_O_4_ superparticles (SPs), loaded with imiquimod (R837 a Toll-like receptor 7 agonist)	4T1 triple-negative human breast cancer cells	Efficient photothermal ablation of 4T1 cells by apoptosis/necrosis upon PTT irradiation, efficient delivery of R837 in vivo against primary tumors to enhance immune response [344]
Fe_3_O_4_@PDA SPs	HeLa human cervical cancer cell line, mice bearing tumor (HeLa)	Biocompatible, increased efficacy of photothermal therapy against tumors in vivo [345]
IONPs—loaded with curcumin and coated with dextran CUR/DEX/Fe_3_O_4_-NPs	MCF-7 human breast cancer	Decreased cell viability in a dose and time related manner [346]
SPIONS functionalized with SDS and loaded with curcumin and coated with chitosan SPIONs-SDS-CU-CHIT	HeLAa human cervical cancer	Decreased viability in a dose and time related manner related to drug release in the medium [347]

**Table 2:** Combined in vitro effects of iron oxide nanoparticles with and without magnetic hyperthermia with chemotherapy.

PACLITAXEL		
Nanoparticles	Model	Main Results
Multifunctional mesoporous silica nanoparticles (SPIONs) Surface modifications: Fluorescent dye molecules/Hydrophilic groups/Cancer-specific targeting ligands—folate (FA); Drugs: Camptothecin (CPT)/Paclitaxel (PTX)	Human pancreatic cancer cell lines: PANC-1, BxPC3, human foreskin fibroblasts (HFF) as control	Selective cytotoxicity; dual imaging capability; targeted drug delivery through ligands (FA) [229]
SPIONs coated with lauric acid and human serum albumin as carriers for paclitaxel (SPION-LA-HSA-Ptx)	Human breast cancer cell lines (T-47D, BT-474, MCF-7, and MDA-MB-231 cells)	High potential for magnetically targeted drug delivery in breast cancer Similar effects on human breast cancer as PTX alone [234]
SPION@Cs-PTX-PEG-FA SPIONs with paclitaxel (PTX)-loaded chitosan (Cs), polyethylene glycol (PEG), and receptors that target folate (FA)	WEHI-164: Mouse fibrosarcoma; MEF: Mouse embryonic fibroblast (normal) cell line	High nanoparticle stability, selective uptake, reduced systemic toxicity due to the FA receptors, apoptosis of cancer cells [238]
Fe_3_O_4_@LaF_3_:Ce^3+^,Tb^3+^/chi NPs bonded with Paclitaxel (PTX)	A549 human lung cancer cell line	Increased cell toxicity compared to free paclitaxel; efficient imaging (MRI and fluorescence imaging); reduced side effects [239]
MNPs coated with an amphiphilic polymer containing disulfide linkages (Hyaluronic Acid–disulfide bond–Polylactic Acid, HA-SS-PLA), loaded with PTX	HeLa cells human cervical cancer cell line)	Targeted delivery, through magnetism and redox response; improved cytotoxicity, and biocompatibility [348]
DOXORUBICIN		
A54 peptide-functionalized poly(lactic-co-glycolic acid)-grafted dextran (A54-Dex-PLGA) micelles with DOX/SPIO	BEL-7402, HepG2 hepatic cancer	MNPs easy synthesis of SPIONs, low off-target distribution and toxicity; controlled drug release; dual imaging/therapy function [236]
Electro-spun fibers co-loaded with magnetic IONPs, cubic shaped loaded with doxorubicin	Mouse embryonic fibroblast cell line (NIH 3 T3 cells), DOX-sensitive HeLa-WT cervical cancer cells and the DOX-resistant MCF7 breast cancer cells	Hyperthermia combined with enhanced diffusion of DOX—effective oncotherapy [239]
Doxorubicin-loaded IONPs with surface coatings like trimethoxysilylpropyl-ethylenediamine triacetic acid (EDT)	MDCK-MDR1-GBM co-culture model	High DOX penetration through BBB; effective magnetic targeting and reduced systemic toxicity; possibly overcoming MDR cancer cells [241]
Fe_3_O_4_@MnO_2_@PPy nanocomposite loaded with DOX; Fe_3_O_4_ (Iron oxide) core; MnO_2_ (Manganese dioxide) shell; PPy (Polypyrrole) outer layer	Human hepatoma (HepG2)	Good magnetic targeting delivery and enhanced cancer toxicity improved PDT/photothermal therapy (PTT) reduced side effects and better tolerance to hypoxia induced by PDT/PTT [247]
IONP DOX: PEG-coated, doxorubicin-loaded nanoparticles	HeLa cells (human cervical cancer cell line)	Delivery of DOX directly into the cytoplasm trough macro pinocytosis and endocytosis; high biocompatibility [252]
PEG-coated Fe_3_O_4_ luteinizing hormone-releasing hormone (LHRH) ligand containing doxorubicin	A549 and MCF-7 cancer cells	Theranostic NP formulation using LHRH ligand with individual chemotherapy and thermotherapy, effective on both cell lines [349]
OTHERS		
Magnetic IONPs/temozolomide	SD3, G-16, G-302, GL-261 cell lines	Combined hyperthermia using IONPs with temozolomide and radiation showed synergistic anti-glioblastoma effects [208]
SPIONs- PLGA core/poly(N-isopropylacrylamide)-carboxymethyl chitosan shell with NU7441/Gemcitabine	A549 and H460 lung cancer cells	Approach for simultaneous radiotherapy and chemotherapy, Folate receptor targeting increased specific uptake [248]
SPIONs (PVA/LDH-coated and PEG/LDH-coated) with Sorafenib	HepG2 human hepatoma/3T3 mouse fibroblast cell line	Strong SP behavior; enhanced anticancer activity and selectivity; minimal side effects [249]
Magnetic-core silica nanoparticles with nano valves and loaded with cucurbituril	MDA-MB-231 breast cancer cells	Targeted delivery using a nano valve system and hyperthermia [350]
Fe-NP2 coated with PEI conjugated with cisplatin (IV) prodrug	Human ovarian carcinoma A2780 cells/cisplatin-resistant A2780DDP cells	Efficient drug delivery overcoming cisplatin resistance through unique internalization pathway of NPs/increased production of ROS [351]
Phospholipid-modified Pt(IV) prodrug-loaded IONP-filled micelles	B16-F10 melanoma cells	Redox-triggered release of cisplatin, ferroptosis of melanoma cells, lower concentration threshold, lymphatic delivery [352]
Nanoflowers MoS_2_@Fe_3_O_4_- loaded with ICG/Pt(IV) indocyanine green (ICG) and platinum (IV) prodrugs {c,c,t-Pt(NH_3_)_2_Cl_2_(OOCCH_2_CH_2_COOH)_2_}	I.929 fibroblasts, HeLa, H22 tumor-bearing Balb/c mice	Biocompatible, theranostics bioimaging capabilities and laser-induced cytotoxicity [353]
Fe(Salen) nanoparticles with μ-oxo N,N′-bis (salicylidene) ethylene diamine	tongue cancer VX2 (rabbit), HSC-3 (human), and OSC-19 (human)	Hyperthermia-guided, temperature stable cytotoxic effects, even at low concentrations [354]

## 6. Conclusions and Future Perspectives

Although SPIONs are widely regarded as biocompatible and clinically translatable, their safe application requires a nuanced understanding of the parameters influencing toxicity. Optimization of surface coatings, control of administered dose and core diameter, and careful design of external stimuli (e.g., magnetic fields) are essential to mitigate adverse effects. Further research, particularly on long-term biodistribution and chronic toxicity, is warranted to ensure their safe and effective use in clinical settings. In the synthesis of IONPs all the factors that interfere with the treatment, factors related to the MNPs, to targeted tissue, to the systemic body responses, to the environmental electromagnetic field, etc., must be considered. MH’s integration with other therapies—particularly immunotherapy, chemotherapy, and smart carriers—marks a shift from single-modality treatment to multifunctional platforms. With growing preclinical validation and early clinical success, IONP-based MH is positioned as a powerful adjunct strategy in cancer therapy, offering both targeted thermal control and synergistic therapeutic potential. MNPs are emerging as promising agents for enhancing the efficacy of cancer treatment. Among the innovative approaches involving MNPs, MH stands out due to its minimally invasive nature, ability to penetrate deep tissues, and potential to selectively induce various cell death pathways—including apoptosis, ferroptosis, necrosis and pyroptosis. MH allows for localized thermal ablation of tumor tissues, minimizing damage to surrounding healthy tissues. Despite numerous preclinical studies demonstrating the therapeutic potential of MH, its clinical translation remains limited. While MH has been approved for the treatment of recurrent glioblastoma, its safety and effectiveness for other types of malignancies still require validation through comprehensive clinical trials. Consequently, the transition of MH from an experimental therapeutic platform to a widely accepted clinical modality—used in conjunction with surgery, radiotherapy, and chemotherapy—will depend on future advancements in interdisciplinary materials science and the development of intelligent, adaptive treatment systems.

Significant progress was made in tailoring the intrinsic properties of MNPs, such as magnetic responsiveness, biocompatibility, and surface functionality. However, further refinement is needed to optimize their performance under clinically relevant AMF conditions. Standardization of experimental parameters—such as MNP concentration, exposure duration, AMF strength, and route of administration—is essential to ensure reproducibility and facilitate clinical translation.

Many MH studies were conducted on in vitro 2D models, containing tumor cells *w*/*wt* co-cultured stromal and/or vascular cells. The 2D models present certain advantages such as easy maintenance, reliability of the results, they usually involve human cell lines, and are suitable for toxicity screening of MNPs, researching mechanisms of cell death induction or tumor escape but they lack the complex spatial tumor structure. There is a great need for better tumor models that match the clinical scenario, particularly the tumor–stroma–immune system interactions, which is the key to understanding the mechanisms of tumor destruction generated by MH as a single therapy or combined with other options, such as radiotherapy, immunotherapy and/or chemotherapy. In view of this, the most reliable models, so far, have been developed in vivo, on lab animals, particularly rodents. There are, however, many limitations related to animal physiology, the ability to generate a certain type of tumor or to the ethical and financial considerations.

Recent advancements in the development of in vitro 3D models, such as the spheroids, organoids, microfluidics, and the possibility of bioprinting creates opportunities for the design of human tumor models, that can comprise multiple human cell types grown on organic 3D scaffolds like collagen, Matrigel, and others. These models can develop ingrown tumor vascularization and by adding immune cells can generate a certain immune response. Therefore, the 3D models can represent a step forward in standardized testing of MH to generate more reliable data in the preclinical testing. However, the creation of these models requires time, knowledge, and financial resources, and reproducibility can also be an issue, depending on the donor, media, and reactives used for the model creation.

Future research should prioritize the design of multifunctional MNPs capable of integrating diagnostic and therapeutic modalities (so-called “theranostics”) into a single nanoplatform. Such platforms would allow for precise in vivo tumor localization, real-time imaging, and patient-specific treatment, contributing to the realization of personalized oncology. A key challenge remains the efficient targeting and accumulation of MNPs at tumor sites. Passive targeting via the enhanced permeability and retention (EPR) effect often results in significant off-target deposition, particularly in organs such as the liver, spleen, and kidneys. To overcome this, advanced targeting strategies—including ligand-mediated active targeting and magnetic field-assisted navigation—should be further explored. In parallel, the development of next-generation imaging and tracking technologies will be critical to monitor in vivo distribution and enhance tumor-specific accumulation of MNPs via systemic administration. Looking forward, MNPs may also play a pivotal role in preventing metastasis. One conceptual application involves engineering MNPs to circulate within the bloodstream and capture circulating tumor cells, directing them toward an implanted magnetic device for sequestration and removal—a novel approach for metastasis interception. Combination of MH and immunotherapy has the potential to inhibit the suppressive effect of the tumor cytokines, particularly towards stroma infiltrating macrophages and trigger a phenotype switch. Combined with enhanced tumor antigen release by MH-induced cell killing, it can lead to an effective immune response against tumor antigens, leading to local and distant tumor destruction. Additionally, MNPs could be engineered to facilitate non-invasive biopsies of tumors that are otherwise inaccessible through conventional methods, offering new possibilities for early diagnosis and molecular profiling. In cancer immunotherapy, MNPs may serve as potent carriers for vaccine delivery, improving antigen presentation and immune activation. The continued convergence of nanotechnology, immunology, and bioengineering will likely unlock new therapeutic paradigms that exploit the full potential of MNPs in precision oncology.

## Data Availability

Data is contained within the article.
